# Improving mentalizing deficits in older age with region-specific transcranial direct current stimulation

**DOI:** 10.1007/s11357-024-01206-z

**Published:** 2024-06-15

**Authors:** Alexander Lischke, Rike Pahnke, Anna Mäder, Andrew K. Martin, Marcus Meinzer

**Affiliations:** 1 Department of Psychology, Medical School Hamburg, Am Kaierkai 1, 20457 Hamburg, Germany; 2 Institute of Clinical Psychology and Psychotherapy, Medical School Hamburg, Hamburg, Germany; 3 Institute of Sports Science, University of Rostock, Rostock, Germany; 4 Department of Neurology, University Medicine Greifswald, Greifswald, Germany; 5 Department of Psychology, University of Kent, Canterbury, UK; 6 Kent and Medway Medical School, University of Kent, Canterbury, UK

**Keywords:** Transcranial direct current stimulation, Mindreading, Temporo-parietal junction, Aging

## Abstract

Older adults have difficulties to detect the intentions, thoughts, and feelings of others, indicating an age-associated decline of socio-cognitive abilities that are known as “mentalizing”. These deficits in mental state recognition are driven by neurofunctional alterations in brain regions that are implicated in mentalizing, such as the right temporo-parietal junction (rTPJ) and the dorso-medial prefrontal cortex (dmPFC). We tested whether focal transcranial current stimulation (tDCS) of the rTPJ and dmPFC has the potential to eliminate mentalizing deficits in older adults. Mentalizing deficits were assessed with a novel mindreading task that required the recognition of mental states in child faces. Older adults (*n* = 60) performed worse than younger adults (*n* = 30) on the mindreading task, indicating age-dependent deficits in mental state recognition. These mentalizing deficits were ameliorated in older adults who received sham-controlled andodal tDCS over the rTPJ (*n* = 30) but remained unchanged in older adults who received sham-controlled andodal tDCS over the dmPFC (*n* = 30). We, thus, showed for the first time that anodal tDCS over the rTPJ has the potential to remediate age-dependent mentalizing deficits in a region-specific way. This provides a rationale for exploring stimulation-based interventions targeting mentalizing deficits in older age.

**Supplementary Information:**

The online version contains supplementary material available at 10.1007/s11357-024-01206-z.

## Introduction

Older adults often have more difficulties to detect the intentions, thoughts, and feelings of others than younger adults [1], indicating an age-associated decline of socio-cognitive abilities that have been subsumed under the term “mentalizing” [2]. Typical examples of these abilities are perspective-taking, empathetic accuracy or emotion recognition [3]. Deficits in mentalizing are common in normal aging and even more pronounced in pathological aging conditions, like, for example, fronto-temporal dementia or Alzheimer’s disease [4]. These deficits may have dramatic real-life consequences for the mental and physical health of older adults [5], indicating a need for interventions that target mentalizing deficits in older age.

Focal transcranial direct current stimulation (tDCS) may be particularly suited for this purpose because it allows a targeted modulation of neural networks in a region- or task-specific way [6–8]. Moreover, tDCS has already been shown to improve mental state recognition in younger adults by modulating activity in brain regions of the mentalizing network [9]. This network comprises several core hubs for socio-cognitive functions, including the right temporo-parietal junction (rTPJ) and the dorso-medial prefrontal cortex (dmPFC) [10, 11]. These regions are, therefore, promising targets for stimulation-based interventions to ameliorate mentalizing deficits in older adults.

The present proof-of-principle study explored the potential of rTPJ and dmPFC stimulation for the improvement of mentalizing deficits in older age. Mentalizing deficits were assessed with a novel mindreading task in older and younger adults (Reading the Mind in the Eyes of Children Test, RME-C-T; [12]). The mindreading task required the recognition of mental states in child faces. In contrast to other mentalizing tasks (e.g., false-belief or rational-action tasks), the mindreading task does not draw heavily on executive functions [13]. Differences in task performance between young and older adults can, therefore, be attributed more to age-dependent mentalizing deficits. Stimulation effects on age-dependent mentalizing deficits were investigated with focal tDCS that constrains the current flow to circumscribed brain regions, an approach allowing region-specific modulation of socio-cognitive functions [7, 8]. Because performance on mindreading tasks depends more on rTPJ than dmPFC functioning [11], more pronounced changes in task performance were expected during rTPJ than dmPFC stimulation.

## Methods

### Participants

Sixty-one older adults and thirty healthy younger adults were initially recruited as participants for the study, which was approved by the ethics committee of the University of Greifswald. To be included in the study, participants had to be native German speakers, to be aged between 18 and 35 (younger adults) or 60 and 75 (older adults) years, and to be unimpaired in audio-visual abilities. Participants with mental or neurological conditions known to affect mentalizing processes (e.g., head trauma or mental disorders) were excluded. Participants with contraindications for electric brain stimulation (e.g., cardiac pacemakers or cochlea implants) were also excluded. Inclusion and exclusion of participants was determined during a phone interview that was based on current guidelines for brain stimulation studies [14]. The interview comprised questions asking for demographic information, audio-visual impairments, mental and neurological conditions, and electric brain stimulation contraindications (see Supplemental Material S1). All participants who were included in the study provided written informed consent for participation and were compensated with 30 €.

### Procedure

Following the phone interview, all eligible participants were invited for a baseline assessment of their demographic, cognitive, and socio-cognitive characteristics. Demographic characteristics (age, sex, years of education) were assessed with an in-house questionnaire, cognitive characteristics (neuropsychological impairments) were assessed with a screening test (MMSE; [15]), and socio-cognitive characteristics (empathetic abilities) were assessed with a self-report questionnaire (IRI-SPF; [16]). Participants also completed a short version of the mindreading task (RME-C-T [12],) for a baseline assessment of their mentalizing abilities. Please note that the data of three older participants and one younger participant were lost due to experimenter error (i.e., overwriting of data files, failure to save data files). Baseline data was, therefore, only available for 58 out of 61 older and 29 out of 30 younger participants (see Table 1).

**Table 1:** Baseline differences in participant characteristics and mindreading

	YNG^a^ (*n* = 29)		OLD-TPJ^a^ (*n* = 29)		OLD-PFC^a^ (*n* = 29)	
	*M*	*SD*	*M*	*SD*	*M*	*SD*
Age (years)	22.79	2.32	69.22	3.80	68.93	3.83
Education (years)	15.69	1.72	16.30	2.76	15.86	2.19
Cognitive status (MMSE)	29.76	0.70	29.22	0.75	29.14	0.95
Empathetic abilities^b^ (IRI-SPF)	46.17	6.74	44.41	7.58	42.00	8.72
Mentalizing – Recognition (proportion, RME-C-T-B)	0.76	0.08	0.63	0.09	0.62	0.10
Mentalizing – Speed (seconds, RME-C-T-B)	4.54	1.35	6.58	2.11	6.27	1.32

YNG: younger adults, OLD-PFD: older adults with focal tDCS over the dmPFC, OLD-TPJ: older adults with focal tDCS over the rTPJ, MMSE: Mini Mental Status Test [15], IRI-SPF: Interpersonal Reactivity Index – German Version [16], RME-C-T-B: Reading the Mind in the Eyes of Children – Baseline version [12]

^a^Due to experimenter error data of one YNG participant, two OLD-TPJ participants, and one OLD-PFC participant was lost

^b^Data was missing for two OLD-TPJ participants

Older participants were additionally invited to an experimental investigation that was scheduled four weeks after the baseline assessment. The experimental investigation explored the therapeutic potential of sham-controlled tDCS for the improvement of age-dependent mentalizing deficits that had been revealed during the baseline assessment. To this end, two experimental groups were formed that were stratified by age and sex: 31 older participants received sham-controlled tDCS over the rTPJ (OLD-TPJ), and 30 older participants received sham-controlled tDCS over the dmPFC (OLD-PFC). Each group of participants completed two cross-over sessions that were scheduled one week apart. On each session, an extended version of mindreading task (RME-C-T; [12]) was performed under active (anodal) or sham stimulation of the respective target site (rTPJ, dmPFC). Anodal and sham stimulation were counter-balanced across sessions and experimental groups. Twenty-nine of the younger participants, who had completed the extended version of the mindreading task (RME-C-T; [12]) without concurrent tDCS, served as a control group (YNG). By comparing mindreading performance between the control and experimental groups, it was possible (a) to assess the degree of mentalizing deficits in older adults under sham tDCS and (b) to determine whether anodal tDCS is capable of restoring mentalizing abilities in older participants to the level of younger participants. Please note that data of 5 out of 61 older participants had to be excluded due to experimenter error (i.e., overwriting of data files, misplacement of electrodes). Valid experimental data was, thus, only available for 28 OLD-PFC and 28 OLD-TPJ participants. These participants did not differ in age, gender distribution, or scheduling of the baseline and cross-over sessions (see Supplementary Material S2).

### Task

**Fig. 1:**
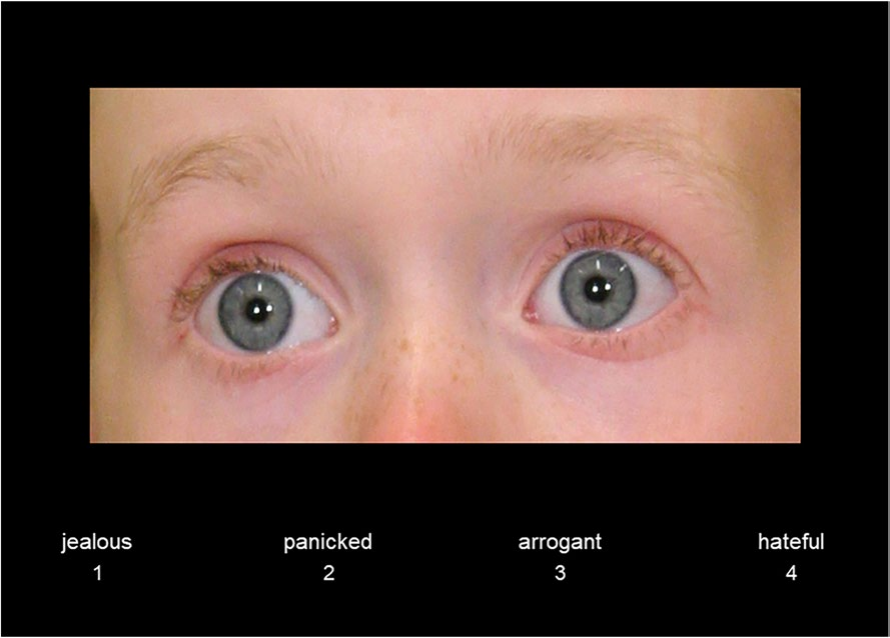
Example of the Reading the Mind in the Eyes of Children Test (RME-C-T; [12]). There are four different mental states (3 distractor labels, 1 target label) that could be expressed by the eye region of the child. Participants had to identify the expressed state by selecting the correct label via a key press on a marked keyboard (panicked)

Participants’ mentalizing abilities were assessed with the Reading the Mind in the Eyes of Children Test (RME-C-T; [12]), a novel mindreading task that was based on the original Reading the Mind in the Eyes Test (RME-T [17]).^1^ The RME-C-T required participants to infer mental states from the eye region of child faces (e.g., shame, regret, gratitude). The eye regions were presented with labels describing four different mental states, three distractor states, and one target state (see Fig. 1). By pressing marked keys on a keyboard (4AFC), participants had to identify the mental states that were expressed by the eye region (see Fig. 1). All eye regions were derived from children who had been trained to express the mental states (see [12] for details).

Two different task versions were used to assess participants’ mentalizing abilities during the baseline assessment and experimental investigation. Both versions involved the presentation of eye regions that had been selected from a validated pool of 351 eye regions (see [12] for details). By selecting different but comparable eye regions from this item pool, two non-overlapping item sets were created that were similar in item content and item difficulty. Each item set comprised practice items to familiarize participants with the task procedure and test items to assess participants’ task performance. A larger item set was used for the experimental version (RME-C-T-E, 51 items) than for the baseline version (RME-C-T-B, 27 items) to maximize the power for the detection of simulation-induced changes in participants’ task performance. Each participant viewed the items of a particular task version in a randomized order during the baseline and experimental sessions.

All task versions were run on a Windows laptop that was equipped with a 22-inch monitor for stimulus presentation and a keyboard for response registration. For statistical analyses, the proportion of correctly recognized mental states (recognition accuracy) and the corresponding response latencies for correctly recognized mental states (recognition speed) were determined. Response latencies were corrected for outliers to account for the high variability in reaction times.^2^

### Transcranial direct current stimulation

A one-channel, battery-driven stimulator was used for transcranial direct current stimulation (neuroConn DC-Stimulator Plus, neuroCare, Munich, Germany). A small (2.5 cm diameter) center anode delivered the current (1 mA) to different target regions (rTPJ, dmPFC). A ring-shaped cathode was placed equidistantly around the central anode (dmPFC: inner/outer diameter, 9.2/11.5 cm; rTPJ: inner/outer diameter, 7.5/9 cm). Please note that the rTPJ cathode was slightly smaller than the dmPFC cathode because of anatomical constraints (i.e., to avoid overlap of the cathode with the right ear). The current modelling for this set-up has demonstrated focal stimulation of brain activity at the respective sites and peak electrical field strength (0.59 V/m) was identified at MNI coordinates 60/54/13 for rTPJ stimulation and 0/54/33 for dmPFC stimulation [19]. Safety has also been demonstrated for this set-up [7].

Scalp positions of the anodes were identified using the 10–20 international EEG system. The dmPFC site was located at 15% of the distance from Fz to Fpz, whereas the rTPJ site was located at CP6 [8]. The electrodes were attached with an adhesive conductive paste (Ten20, Weaver and Company, Aurora, USA) and securely held in place with an EEG cap. At both sites, the current was initially ramped up to 1 mA (8 s). In the sham condition, it was ramped down (5 s) after 40 s of active stimulation. In the anodal condition, the current was maintained at 1 mA for 20 min prior to ramping down. This procedure has been shown to result in effective participant blinding [7, 8, 19, 20]. Experimenter blinding was achieved by using the “study mode” of the stimulator (i.e., a pre-assigned code triggered the different stimulation conditions).

### Statistical analysis

One-way ANOVAs were used to investigate whether older participants receiving stimulation over the rTPJ (OLD-TPJ) or the dmPFC (OLD-PFC) differed from younger participants (YNG) in their demographic (age, years of education), cognitive (neuropsychological impairments), and socio-cognitive (empathy) characteristics. A similar ANOVA with follow-up tests was conducted to investigate baseline differences in mindreading performance (recognition accuracy, recognition speed). Stimulation-induced differences in OLD-TPJ and OLD-PFC participants’ mindreading performance were analyzed with two-way mixed-design ANOVAs (Site × Stimulation) and follow-up tests. These ANOVAs and follow-up tests were additionally adjusted for baseline differences in mindreading performance and socio-cognitive characteristics to assess the robustness of stimulation-induced differences in OLD-TPJ and OLD-PFC participants’ mindreading performance (see Supplementary Material S3). Independent *t*-tests were used to compare simulation-induced differences in OLD-TPJ and OLD-PFC participants’ mindreading performance with YNG participants mindreading performance. Stimulus-induced side effects were analyzed with two-way mixed-design ANOVAs (Site × Stimulation).

Significance levels for all analyses were set to *α* < 0.05. To facilitate the interpretation of the results, we also report effect size measures (*d, η*^*2*^_*p*_). Analyses were carried out with SPSS 27 (IBM Corp., Armonk, NY, USA) and JASP Version 0.17.1 (https://jasp-stats.org/).

## Results

### Participant characteristics

Except for differences in age and cognitive status, there were no further differences in demographic, cognitive or socio-cognitive characteristics between older and younger participants [age, *F*(2,84) = 1641.442, *p* < 0.001, *η*^*2*^_*p*_ = 0.975; OLD-TPJ vs. OLD-PFC, *p* = 1.00; OLD-TPJ vs. YNG, *p* < 0.001; OLD-TPJ vs YNG, *p* < 0.001; years of education, *F*(2,82) = 0.451, *p* = 0.638, *η*^*2*^_*p*_ = 0.011; empathetic abilities, *F*(2,82) = 2.131, *p* = 0.125, *η*^*2*^_*p*_ = 0.049; see Table 1]. YNG participants’ scored higher on the MMSE than OLD-TPJ and OLD-PFC participants, who showed comparable MMSE scores [MMSE, *F*(2,82) = 4.737, *p* = 0.011, *η*^*2*^_*p*_ = 0.101; OLD-TPJ vs. OLD-PFC, *p* = 1.00; OLD-TPJ vs. YNG, *p* = 0.044; OLD-PFC vs. YNG, *p* = 0.019]. All MMSE scores were within the normal range of the respective age groups [15], indicating intact cognitive functioning in all participants.

### Baseline differences in mindreading

**Fig. 2:**
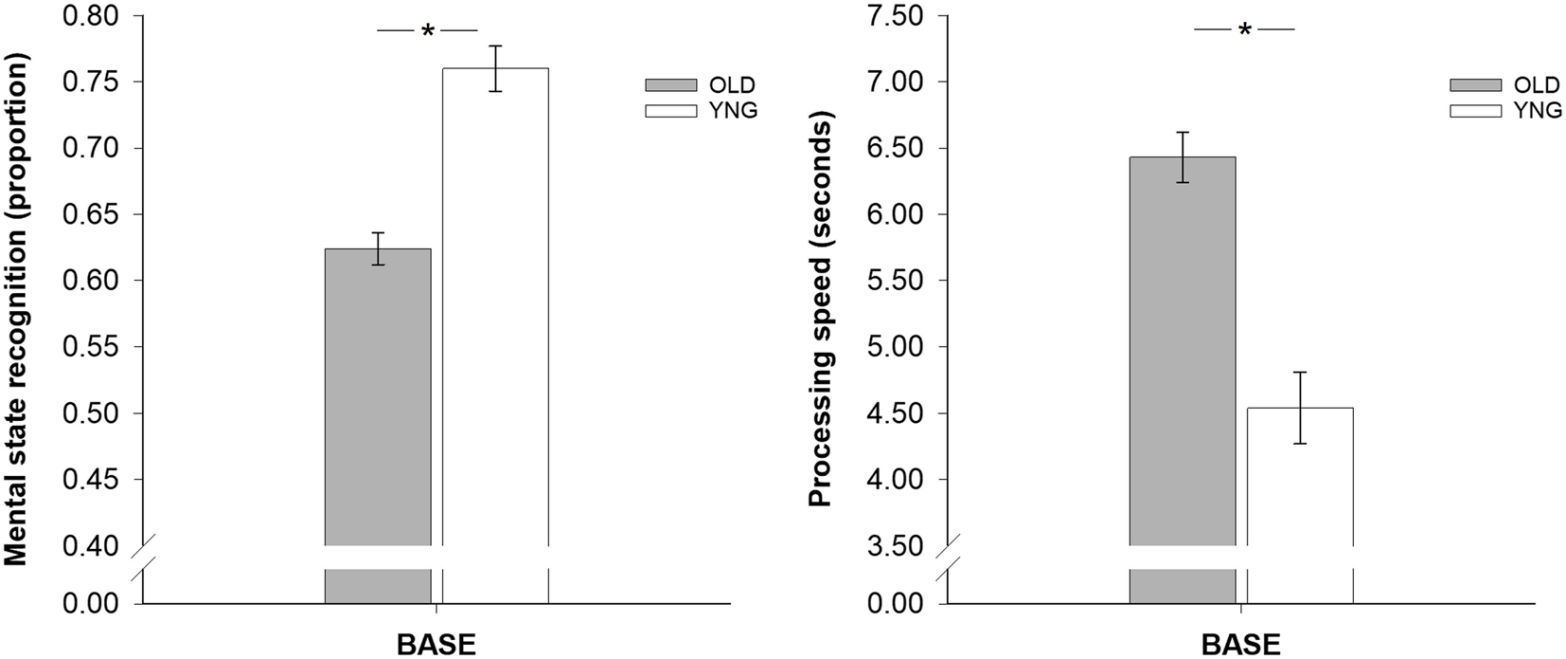
Barplots demonstrating baseline differences in mindreading performance (*left:* recognition accuracy, *right:* recognition speed) between younger adults (YNG, white bar) and older adults (OLD, gray bar). Bars represent *M* ± *SEM*. *p ≤ .05

There were age-dependent baseline differences in recognition accuracy between younger and older participants [*F*(2,84) = 22.22, *p* < 0.001, *η*^*2*^_*p*_ = 0.346; see Table 1 and Fig. 2]: YNG participants were more accurate in mental state recognition than OLD-TPJ and OLD-PFC participants [YNG vs. OLD-TPJ, *p* < 0.001, *d* = 1.55; YNG vs. OLD-PFC, *p* = 0.001, *d* = 1.56]. The OLD-TPJ and OLD-PFC participants were comparable in recognition accuracy [OLD-TPJ vs. OLD-PFC, *p* = 1.00, *d* = 0.15]. A similar pattern of baseline differences between younger and older participants was found for recognition speed [*F*(2,84) = 16.58, *p* < 0.001, *η*^*2*^_*p*_ = 0.283; see Fig. 2]: YNG participants were faster in mental state recognition than OLD-TPJ and OLD-PFC participants [YNG vs. OLD-TPJ, *p* < 0.001, *d* = 1.55; YNG vs. OLD-PFC, *p* < 0.001, *d* = 1.56] who did not differ in recognition speed [OLD-TPJ vs. OLD-PFC, *p* = 1.00, *d* = 0.15].

### Simulation-induced differences in mindreading

**Fig. 3:**
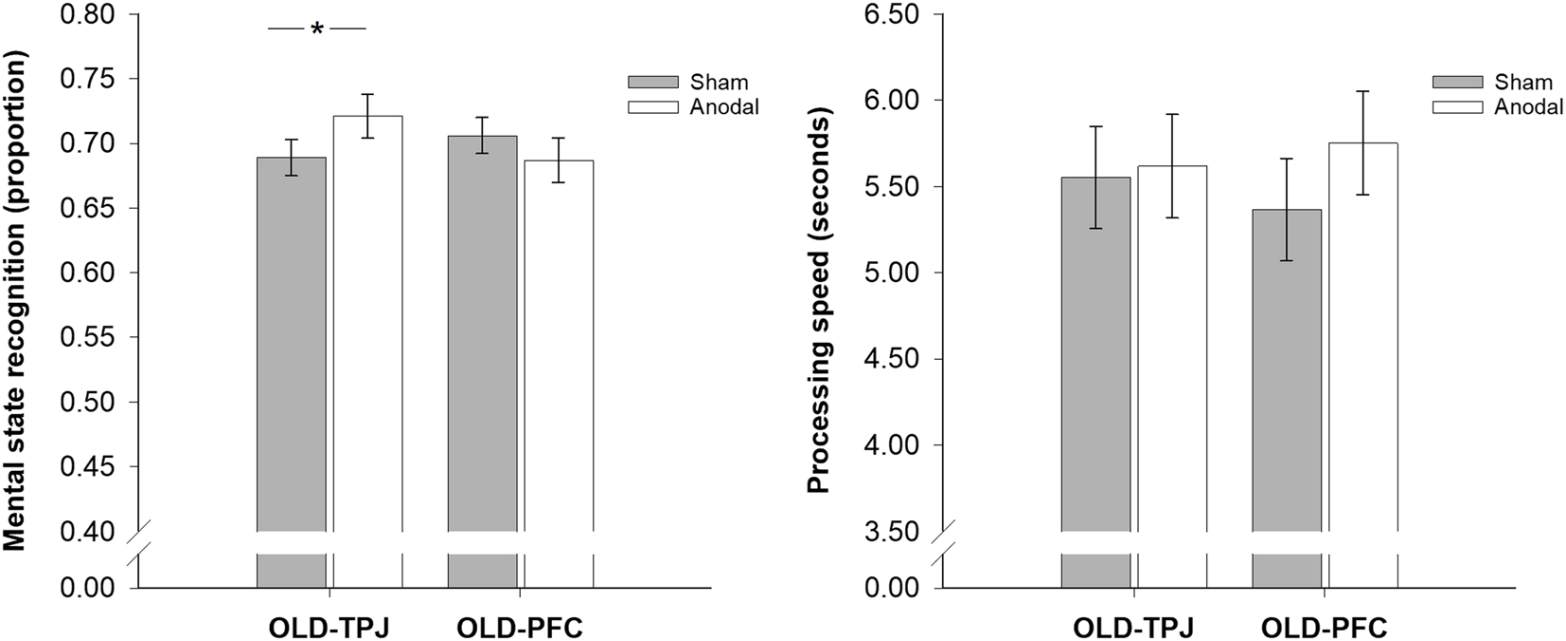
Barplots demonstrating stimulation-induced differences in mindreading performance (*left:* recognition accuracy; *right:* recognition speed) between older adults that received sham (gray bars) or anodal (white bars) tDCS over the rTPJ (OLD-TPJ) or dmPFC (OLD-PFC). Bars represent *M* ± *SEM*. *p ≤ .05

Depending on the stimulation site, anodal stimulation had different effects on recognition accuracy than sham stimulation in older participants [Site, *F*(1,54) = 0.19, *p* = 0.668, *η*^*2*^_*p*_ = 0.003; Stimulation, *F*(1,54) = 0.60, *p* = 0.442, *η*^*2*^_*p*_ = 0.011; Site × Stimulation, *F*(1,54) = 9.07, *p* = 0.004, *η*^*2*^_*p*_ = 0.144; see Fig. 3]: There were no differences in recognition accuracy between OLD-TPJ and OLD-PFC participants during anodal [OLD-TPJ vs. OLD-PFC, *p* = 0.404, *d* = 0.38] or sham [OLD-TPJ vs. OLD-PFC, *p* = 0.156, *d* = 0.23] stimulation. OLD-TPJ participants were, however, more accurate in mental state recognition during anodal than sham stimulation [anodal vs. sham, *p* = 0.010; *d* = 0.59]. OLD-PFC participants, on the contrary, did not differ in recognition accuracy during anodal and sham stimulation [anodal vs. sham, *p* = 0.119; *d* = 0.28]. Anodal stimulation over the rTPJ, thus, led to a site-specific improvement of older participants’ recognition accuracy, even when adjusting for baseline differences in mental state recognition or empathetic abilities (see Supplementary Material S3).

However, anodal stimulation over the rTPJ did not abolish the age-dependent difference in recognition accuracy between OLD-TPJ and YNG participants: OLD-TPJ participants receiving anodal stimulation were still less accurate in mental state recognition than YNG participants [OLD-TPJ anodal vs. YNG, *t*(55) = 4.11, *p* < 0.001, *d* = 1.09; see Supplementary Figure S1]. YNG participants were generally more accurate in mental state recognition than OLD-TPJ or OLD-PFC participants, irrespective of stimulation condition or stimulation site [OLD-TPJ sham vs. YNG, *t*(55) = 6.55, *p* < 0.001, *d* = 1.73; OLD-PFC anodal vs. YNG, *t*(55) = 5.97, *p* < 0.001, *d* = 1.58; OLD-PFC sham vs. YNG, *t*(55) = 5.40, *p* < 0.001, *d* = 1.43; see Supplementary Figure S1].

Anodal stimulation had no effect on older participants’ recognition speed at either stimulation site [Site, *F*(1,54) = 0.005, *p* = 0.946, *η*^*2*^_*p*_ = 0.000; Stimulation, *F*(1,54) = 1.483, *p* = 0.229, *η*^*2*^_*p*_ = 0.027; Site × Stimulation, *F*(1,54) = 0.744, *p* = 0.392, *η*^*2*^_*p*_ = 0.014; see Fig. 3]. There were no differences in recognition speed during anodal or sham stimulation among OLD-TPJ [anodal vs. sham, *p* = 0.803, *d* = 0.05] or OLD-PFC [anodal vs. sham, *p* = 0.147, *d* = 0.28] participants. OLD-TPJ and OLD-PFC participants also did not differ in recognition speed during anodal [OLD-TPJ vs. OLD-PFC, *p* = 0.753, *d* = 0.09] or sham [OLD-TPJ vs. OLD-PFC, *p* = 0.660, *d* = 0.12] stimulation.

Anodal stimulation did not abolish the age-dependent differences in recognition speed among OLD-TPJ and OLD-PFC participants: OLD-TPJ and OLD-PFC participants receiving anodal stimulation were still slower in mental state recognition than YNG participants [OLD-TPJ anodal vs. YNG, *t*(55) = 4.21, *p* < 0.001, *d* = 1.15; OLD-PFC anodal vs. YNG, *t*(55) = 3.36, *p* < 0.001, *d* = 0.89; see Fig. 3 and Supplementary Figure S1]. YNG participants were also faster in mental state recognition than OLD-TPJ and OLD-PFC participants receiving sham stimulation [OLD-TPJ sham vs. YNG, *t*(55) = 3.33, *p* = 0.002, *d* = 0.83; OLD-PFC sham vs. YNG, *t*(55) = 2.83, *p* = 0.007, *d* = 0.75; see Supplementary Figure S1].

### Stimulation-induced side-effects

Adverse effects were only reported by a minority of older participants (see Table 2). However, reports did not differ between OLD-TPJ and OLD-PFC participants after anodal or sham stimulation [Site, all *F*(1,54) < 2.29, all *p* > 0.136, all *η*^*2*^_*p*_ < 0.041; Stimulation, *F*(1,54) < 1.23, all *p* > 0.272, all *η*^*2*^_*p*_ < 0.022; Site x Stimulation, all *F*(1,54) < 3.74, all *p* > 0.058, all *η*^*2*^_*p*_ < 0.065]. The lack of stimulation-induced side effects makes it unlikely that older participants were able to differentiate anodal from sham stimulation based on physical scalp sensations as in previous studies that used identical stimulation protocols and formal blinding assessments [7, 8, 19, 20].

**Table 2:** Stimulation-induced adverse side effects

	OLD-TPJ^a,b^				OLD-PFC^a,b^			
	Sham tDCS (n = 28)		Anodal tDCS (n = 28)		Sham tDCS (n = 28)		Anodal tDCS (n = 28)	
	M	SD	M	SD	M	SD	M	SD
Itching	0.04	0.19	0.11	0.31	0.21	0.63	0.11	0.31
Pain	0.00	0.00	0.11	0.57	0.00	0.00	0.00	0.00
Burning	0.29	0.66	0.29	0.71	0.18	0.48	0.32	0.67
Heat	0.14	0.45	0.25	0.70	0.11	0.31	0.18	0.48
Fatigue	0.07	0.38	0.21	0.57	0.07	0.26	0.07	0.26
Other	0.21	0.42	0.14	0.36	0.18	0.39	0.32	0.55

*OLD-PFC* older adults with focal tDCS over the dmPFC, *OLD-TPJ* older adults with focal tDCS over the rTPJ

^a^Due to participant drop-out data of two OLD-TPJ participants and one OLD-PFC participant was missing

^b^Due to experimenter error data for one OLD-TPJ participant and one OLD-PFC participant was lost

## Discussion

We conducted a proof-of-principle study to investigate whether age-dependent deficits in mental state recognition can be improved with focal tDCS over brain regions that are relevant for mentalizing, the rTPJ and the dmPFC [10, 11]. To this end, we investigated younger and older participants’ mentalizing abilities with a novel mindreading task [12]. The mindreading task required participants to infer mental states from the eye region of child faces [12]. We administered two versions of the mindreading task, a shorter baseline version to demonstrate mentalizing deficits in older participants and a longer experimental version to probe whether mentalizing deficits in older participants improved during sham-controlled stimulaton of the rTPJ or dmPFC.

We first compared younger and older participants’ mentalizing abilities on the baseline version of the mindreading task. Younger participants’ showed a similar mindreading performance on the baseline version as young participants on the original version of the task [12], indicating that even the shorter task version used in our study is well- to investigate differences in mentalizing abilities. Younger participants also outperformed older participants on the baseline version, which is in line with previous studies reporting mentalizing deficits in older participants during the processing of adult faces [21, 22]. Our findings complement and extend these findings by revealing similar mentalizing deficits in older participants during the processing of child faces, indicating a pervasive mentalizing deficit in older age.

Thereafter, we investigated older participants’ mentalizing abilities on the experimental version of the mindreading task during sham-controlled rTPJ and dmPFC stimulation. Older participants receiving sham stimulation showed a similar mindreading performance on the experimental version as on the baseline version, indicating a persistent mentalizing deficit across the different task versions. Older participants receiving anodal stimulation showed a region-specific remediation of this mentalizing deficit: Mindreading performance remained unchanged under anodal dmPFC stimulation but improved under anodal rTPJ stimulation [corresponding effect size, *d* = 0.59]. The stimulation-induced improvement in mindreading performance did not result in a full restoration of older participants’ mentalizing abilities, because their mindreading performance was still worse than younger participants’ mindreading performance on the experimental version. These findings are consistent with previous findings suggesting a stimulation-induced remediation rather than elimination of age-dependent impairments in social and cognitive functioning [23, 24].

Our findings show that mentalizing in older adults involves the rTPJ, which is in line with current models that place the TPJ at the core of a mentalizing network [10, 11]. These models were based on imaging studies that investigated brain activation associated with mindreading tasks [25–27]. Due to the inherent limitations of the imaging approach [28], these studies could only provide *weak* evidence for an involvement of the rTPJ in mentalizing. We, however, provide *stronger* evidence for the rTPJ involvement in mentalizing because our stimulation approach allowed us to modulate brain activity during mentalizing in a direct and focal manner [29]. Our findings, thus, support the current view that mental state recognition depends more on rTPJ than dmPFC functioning [11].

Although our proof-of-principle study highlights the importance of the rTPJ for mentalizing in older adults, we can only speculate about the neural mechanisms causing the age-dependent decline in mental state recognition and the stimulation-induced improvement in mental state recognition. The age-dependent decline in mental state recognition may be driven by age-related alterations in gray matter density and white matter architecture of brain regions that form the mentalizing network [30, 31]. The mentalizing network is centered on the TPJ [11], implying that gray or white matter alterations in any of the interconnected brain regions affect mindreading performance [32]. Gray matter loss in the TJP and white matter loss in TJP-related tracts have already been shown to impair mindreading performance in young adults [33–35]. We, thus, believe that similar gray and white matter alterations may have accounted for older participants’ mentalizing deficits in our study. We think that it is important to view these mentalizing deficits as a result of a disconnection syndrome [13] because TPJ functioning can be better understood from a network than from a modular perspective [36, 37]. The stimulation-induced improvement in mental state recognition may possibly be due to a restoration of connectivity clusters within TPJ-centered networks. Focal stimulation of the TPJ has already been shown to synchronize cluster functioning by enhancing low-frequency oscillations in neural assemblies [38]. We, thus, tentatively assume that stimulation-induced changes in low-frequency oscillations may have accounted for the improved recognition of mental states in older participants.

Despite the plausibility of these assumptions, we have to contend that our proof-of-principle study was limited in several ways. We recruited a similar number of older participants as in previous studies investigating stimulation-induced improvements of social and cognitive functioning in older age [23, 24], but the COVID-19 pandemic complicated the recruitment of additional participants as substitutes for older participants with invalid data [39]. The compromised sample size may have limited the statistical power of our analyses, thereby increasing the chance of false positive findings that were due to random rather than stimulation-induced changes in mindreading performance. In this respect, it is noteworthy that we matched the older participants of the stimulation groups on demographic, cognitive, and socio-cognitive characteristics that are known to modulate in mindreading performance [40]. Older participants of both groups received focal stimulation over meta-analytically defined target regions in a sham-controlled cross-over design during the performance of an age-sensitive mindreading task. The matching procedure and the cross-over design in combination with the administration of an extended task version and focal tDCS helped to control random changes in mindreading performance [41], thereby decreasing the chance of false positive findings. We further decreased the chance of false positive findings by adjusting our analyses for baseline differences in older participants’ mindreading performance and empathetic abilities. All analyses revealed stimulation-induced changes in mindreading performance that correspond to medium-sized effects on the statistical level [42], indicating the robustness of these effects. Nonetheless, we do not know whether these effects are of practical relevance in real life.

Our proof-of-principle study was not designed to address practical issues but to demonstrate that it is in principle possible to improve age-dependent mentalizing deficits with focal tDCS over the rTPJ. Proof-of-principle studies of other methods revealed similar-sized improvements in mindreading performance [43] that motivated further research on the therapeutic potential of these methods [44–46]. We hope that our proof-of-principle study also stimulates studies that further investigate the therapeutic potential of focal tDCS for the treatment of age-dependent mentalizing deficits. These studies should investigate stimulation-induced mentalizing changes in larger samples with more sophisticated methods over longer time intervals. Our study focused on short-term improvements of age-dependent mentalizing deficits following single-session tDCS over the rTPJ. Given the transient nature of single-session stimulation effects [47], it remains to be determined whether multi-session tDCS over the rTPJ results in long-term mentalizing improvements that are of practical relevance for real-life applications.

The neural mechanisms underlying the stimulation-induced improvements of older participants’ mentalizing deficits could not be determined on basis of our study design. Study designs that combine stimulation protocols with imaging protocols are the only way to determine whether focal stimulation of the target site led to circumscribed activity changes in the rTPJ [7]. To validate that these activity changes were associated with mentalizing processes, study designs have to incorporate other tasks alongside mindreading tasks. Otherwise, it cannot be ruled out that improvements in older participants’ mindreading performance were driven by other processes than mentalizing processes (e.g., stimulation-induced changes in visual processing). Processes related to blinding issues should also be better controlled because the present study design lacked a formal assessment of the blinding success. Future studies with more sophisticated designs may help to fully understand why focal stimulation of the rTPJ led to improvements in older participants’ mental state recognition.

Notwithstanding the limitations of our proof-of-principle study, we show for the first time that focal stimulation of the rTPJ improves age-dependent mentalizing deficits. The stimulation-induced improvements in mentalizing suggest a therapeutic potential for the treatment of older adults who have difficulties in mental state recognition. To further explore this therapeutic potential, we have to develop and evaluate stimulation-based treatment approaches that target age-related deficits in mental state recognition. Incorporating stimulation protocols in socio-cognitive training programs may be a promising approach, in particular if the stimulation protocols comprise multi-session tDCS that leads to stronger and longer-lasting changes in socio-cognitive processes than single-session tDCS [47]. Regarding the mechanisms underlying socio-cognitive training programs, it is noteworthy that training-induced mentalizing improvements are accompanied by gray and white matter alterations in brain regions that form the mentalizing network [48]. Gray matter alterations in the rTPJ are the major driver of mentalizing improvements in these training programs [49], indicating training-induced plasticity changes. Stimulating the rTPJ during training sessions may enhance cluster functioning in TPJ-centered networks [38, 47], thereby facilitating plasticity changes that drive mentalizing improvements over the course of the training program. We, thus, believe that incorporating rTPJ-focused stimulation protocols in socio-cognitive training programs represents a promising approach for the treatment of age-dependent mentalizing deficits. We hope that our work stimulates further research in this area.

### Supplementary Information

Below is the link to the electronic supplementary material.

Supplementary file1 (DOCX 309 KB)

## Footnotes

1 Depending on a narrow or broad definition of the mentalizing construct [2, 3], the RME-T can be framed as an emotion recognition or as a mentalizing task. Following recent studies [18] and meta-analyses [10, 11], we describe the RME-T as a mentalizing task.

2 Participants’ reaction times that fell below the Q1-3*IQR or above the Q3 + 3*IQR of their group were replaced by the respective group mean (RME-C-T-B: one YNG participant, one OLD-TPJ participant, and four OLD-PFC participants; RME-C-T-E: two OLD-TPJ participants).
