# Supplementary material for: Improving mentalizing deficits in older age with region-specific transcranial direct current stimulation
Source: GeroScience. 2024 Jun 15;46(5):4111–21. doi: 10.1007/s11357-024-01206-z (PMC11336013; doi:10.1007/s11357-024-01206-z)

**Improving mentalizing deficits in older age with region-specific transcranial direct current stimulation**

**Supplemental Material**

**Alexander Lischke^1,2*^, Rike Pahnke^3^, Anna Mäder^4^, Andrew K. Martin^5,6^, Marcus Meinzer^4^**

^1^Department of Psychology, Medical School Hamburg, Hamburg, Germany
^2^Institute of Clinical Psychology and Psychotherapy, Medical School Hamburg, Hamburg, Germany

^3^Institute of Sports Science, University of Rostock, Rostock, Germany

^4^Department of Neurology, University Medicine Greifswald, Greifswald, Germany
^5^Department of Psychology, University of Kent, Canterbury, United Kingdom
^6^Kent and Medway Medical School, University of Kent, Canterbury, United Kingdom

**Corresponding author:**

Alexander Lischke, Department of Psychology, Medial School Hamburg, Am Kaierkai 1, 20457 Hamburg, Germany. Email: [alexander.lischke@medicalschool-hamburg.de](mailto:alexander.lischke@medicalschool-hamburg.de)

**S1 Phone interview**

Here we provide a translated version of the phone interview that we used to determine inclusion and exclusion of participants.

| **A** | **Phone Interview** | | | |
| --- | --- | --- | --- | --- |
|  | | | | |
| **Date of interview:** | | **___.____.______** | **Name of interviewer:** | **___________________** |
|  | | | | |
| **01.** | **Initial contact** | | | |
| Hello Mr/Mrs _____________________________  I am calling from the Department of Neurology at the University Medicine Greifswald. My name is _____________________________  *If contact via previous study: go to 2*  *If other contact: go to 3*  The reason I am calling is that you have expressed interest in participating in one of our studies. | | | | |
| **02** | **Contact via previous study** | | | |
| … in a previous study you had expressed interest in being contacted about future studies. We are currently looking for participants for a study that investigates social cognition in healthy young and older individuals. Are you interested in participating and may I explain to you what the study is about?  *If no time*: Thank you very much. May I contact you about this study at a later date?  *If yes: arrange alternative date/time: _____________________________________*  *If no:* Do we still have permission to contact you about future studies?  □ yes □ no *(make note, end call)*  *If yes*: Thank you very much (*go to 4*) | | | | |
| **03.** | **Other contact** | | | |
| …you have expressed interest in our study on social cognition in healthy young and older individuals. Would you have a few minutes at the moment, so that I can explain to you what the study is about and ask you a few questions?  *If yes: go to 4*  *If no: arrange alternative date/time: _____________________________________* | | | | |

| **B** | **Phone Interview** |
| --- | --- |
|  | |
| **04.** | **Basic Information** |
| This study is concerned with socio-cognitive processes. This includes abilities that allow us to understand and communicate emotions or to understand the perspective of others. Iin our study, we are interested if these abilities change as we age. Therefore, we will investigate social cognition in healthy young and older individuals. We are also using a non-invasive brain stimulation technique to investigate if we can improve social cognition. This technique is called transcranial direct current stimulation (tDCS). TDCS is a safe technique that has been used for many years to stimulate the human brain and to modulate brain functions. Our hope is that we can use this technique to improve social cognition.  In the study, we will invite you to come to our laboratories three times. During the first time, we will ask you to complete a few questionnaires and conduct a few tests. This will allow us to confirm that you can participate in the study. During two additional visits, you will complete a computerized socio-cognitive task where we ask you to identify mental states from the eyes of children.  We will explain the task in detail at the day of study. During one of these visits, you will also receive real tDCS, during the other one, you will receive placebo tDCS. This will allow us to investigate if the brain stimulation improves your socio-cognitive abilities. We will only tell you after the end of the second visit which type of stimulation you have received at the respective days, so that the study results are not biased.  With your participation, you will help us to understand better how socio-cognitive processes change across the healthy lifespan. It will also help us to find out if tDCS can improve these important processes.  You will receive a financial compensation of 30 € for your participation.  Do you have any questions? | |
| **05.** | **Interest** |
| Are you still interested in participating?  *If no:* Thank you very much, I will take a note (*make not, end call*)  *If yes*: *go to 6* | |

| **C** | **Phone Interview** | |
| --- | --- | --- |
|  | | |
| **06.** | **Health information** | |
| I would like to go through a few questions with you to make sure that it is safe for you to participate in the study.   \|  \| *yes* \| *no* \|  \| \| --- \| --- \| --- \| --- \| \| 01. Do you have any magnetic metal in your body (screws, surgical clips, dentures, cochlea implant, etc.)? \|  \|  \| ______________________ \| \| 02.Do you have a cardiac pacemaker? \|  \|  \| ______________________ \| \| 03. Do you have a heart condition? \|  \|  \| ______________________ \| \| 04. Did you have a stroke, head surgery or other neurological conditions? \|  \|  \| ______________________ \| \| 05. Did you have a traumatic head injury (with/without unconsciousness) \|  \|  \| ______________________ \| \| 06. Do you have a head tumor? \|  \|  \| ______________________ \| \| 07. Do you have seizures or frequent migraines? \|  \|  \| ______________________ \| \| 8. Do you have a psychiatric condition (depression, anxiety, psychosis, etc.)? \|  \|  \| ______________________ \| \| 9. Do you have trouble hearing or visual problems? \|  \|  \| ______________________ \| \| 10. Do you have any other medical conditions? \|  \|  \| ______________________ \| \| 11. Do you have a history of drug/alcohol dependency? \|  \|  \|  \| \| 12. Have you ever noticed memory problems?  If yes (>6 months?) \|  \|  \| ______________________ \| \| Are you concerned about them? \|  \|  \| ______________________ \| \| Have you consulted a doctor? \|  \|  \| ______________________ \| \| 13.Are you currently taking medication? \|  \|  \| ______________________ \| \| 14. Do you have hypertension? \|  \|  \| ______________________ \| \| 15. Do you have Diabetes, Astma or Glaucoma? \|  \|  \| ______________________ \| \| 16.Are you pregnant (Women)? \|  \|  \| ______________________ \| \| 17. Claustrophobia? \|  \|  \| ______________________ \| \| 18. Are you right-handed? \|  \|  \| ______________________ \|   *If eligible*: *go to 7*  *If inneligible*. Thank you very much. Unfortunately, we cannot include you in this particular study. May we contact you again in the future? □ yes □ no *(make note, end call)* | | |
| **D** | **Phone Interview** | |
|  | | |
| **07.** | **Confirmation** | |
| Thank you very much. You can participate in our study.  Do you have any questions?  *Are you still interested in participating?* □ yes □ no  *If no:* Thank you very much for your time. *(make note, end call)*  *If yes:* Thank you very much. I only have a few additional questions (*go to 8*) | | |
|  | | |
| **08.** | **Demographic information** | |
| I have some final questions regarding your demographic characteristics. | | |
| Date of birth:  Sex:  Age (years):  Height (cm);  Weight (in kg):  Native speaker (German) | | ___.____.______  □ male □ female □ other  _____________________________  _____________________________  _____________________________  □ yes □ noe |
| **09.** | **Study information / consent procedures** | |
| *Wants to participate?* □ yes □ no | | |
| For you to be able to participate, we will need your written consent. We will now send you study specific information and a consent form. To do so, we need your postal address and you email address.  Postal address  _______________________________________________________________________________________  _______________________________________________________________________________________  _______________________________________________________________________________________  Email address  _______________________________________________________________________________________  Please read the study specific information and a consent form carefully and send the consent form back to us by using the pre-paid envelope or as scan (email). If you prefer email, please bring the original version with you at the first day of the study.  Once we have received your consent form, we will be in touch about the first appointment.  Do you have any further questions? | | |
|  | | |
| **10.** | **End** | |
| Thank you again for your time and interest in our study. | | |

**S2 Participant characteristics**

Valid experimental data was available for 28 OLD-PFC and 28 OLD-TPJ participants. These participants. These participants did not differ in age [OLD-PFC: *M* = 69.36, *SD* = 4.18, , OLD-PFC: *M* = 69.21, *SD* = 3.90; OLD-TPJ vs. OLD-PFC: *t*(54) = 0.13, *p* = .895, *d* = 0.04] or gender distribution [OLD-PFC: 17 female, 11 male, OLD-PFC: 15 female, 13 male; OLD-TPJ vs. OLD-PFC: *χ^2^*(2) = 0.31, *p* = .857]. There were also no differences regarding the scheduling of the baseline session [OLD-TPJ: *M* = 32.75, *SD* = 51.84, OLD-PFC: *M* = 34.46, *SD* = 101.61; OLD-TPJ vs. OLD-PFC: *t*(54) = 0.08, *p* = .937, *d* = 0.02]or the scheduling of the cross-over sessions [OLD-TPJ: *M* = 7.14, *SD* = 0.35, OLD-PFC: *M* = 7.04, *SD* = 0.18; OLD-TPJ vs. OLD-PFC: *t*(42.50) = 1.48, *p* = .166, *d* = 0.37] between the participants.

**S3 Stimulation-induced differences in mindreading**

Anodal stimulation of the rTPJ improved older participants’ mindreading performance, regardless whether the corresponding analyses were controlled for baseline differences in mental state recognition [RME-C-T-B: Site: *F*(1,50) = 0.41, *p* = .841, *η^2^_p_* = .001; Stimulation: *F*(1,50) = 0.89, p = .350, *η^2^_p_* = .017; Site x Stimulation: *F*(1,50) = 8.54, *p* = .005, *η^2^_p_* = .146; OLD-TPJ anodal vs. OLD-PFC anodal: *p* = .196, *d* = 0.35; OLD-TPJ sham vs. OLD-PFC sham: *p* = .234, *d* = 0.32; OLD-TPJ anodal vs. OLD-TPJ sham: *p* = .029; *d* = 0.61; OLD-PFC anodal vs. OLD-PFC sham: *p* = .064; *d* = 0.37] or empathy [IRI: *F*(1,51) = 0.13, *p* = .910, *η^2^_p_* = .000; Site x Stimulation: *F*(1,51) = 7.43, *p* = .009, *η^2^_p_* = .127; OLD-TPJ anodal vs. OLD-PFC anodal: *p* = .166, *d* = 0.39; OLD-TPJ sham vs. OLD-PFC sham: *p* = .482, *d* = 0.19; OLD-TPJ anodal vs. OLD-TPJ sham: *p* = .029; *d* = 0.54; OLD-PFC anodal vs. OLD-PFC sham: *p* = .125; *d* = 0.28]. Considering that these analyses revealed similar results indicates the robustness of stimulation-induced improvements in age-dependent mindreading performance.

**Figure S1.** Barplots demonstrating stimulation-induced differences in mindreading performance (*upper panel:* recognition accuracy; *lower panel:* recognition speed) between younger adults that received no tDCS (white bars) and older adults that received sham or anodal tDCS over the rTPJ (OLD-TPJ, light-gray bars) or dmPFC (OLD-PFC, dark gray bars). Bars represent *M* ± *SEM*. * *p* ≤ .001.


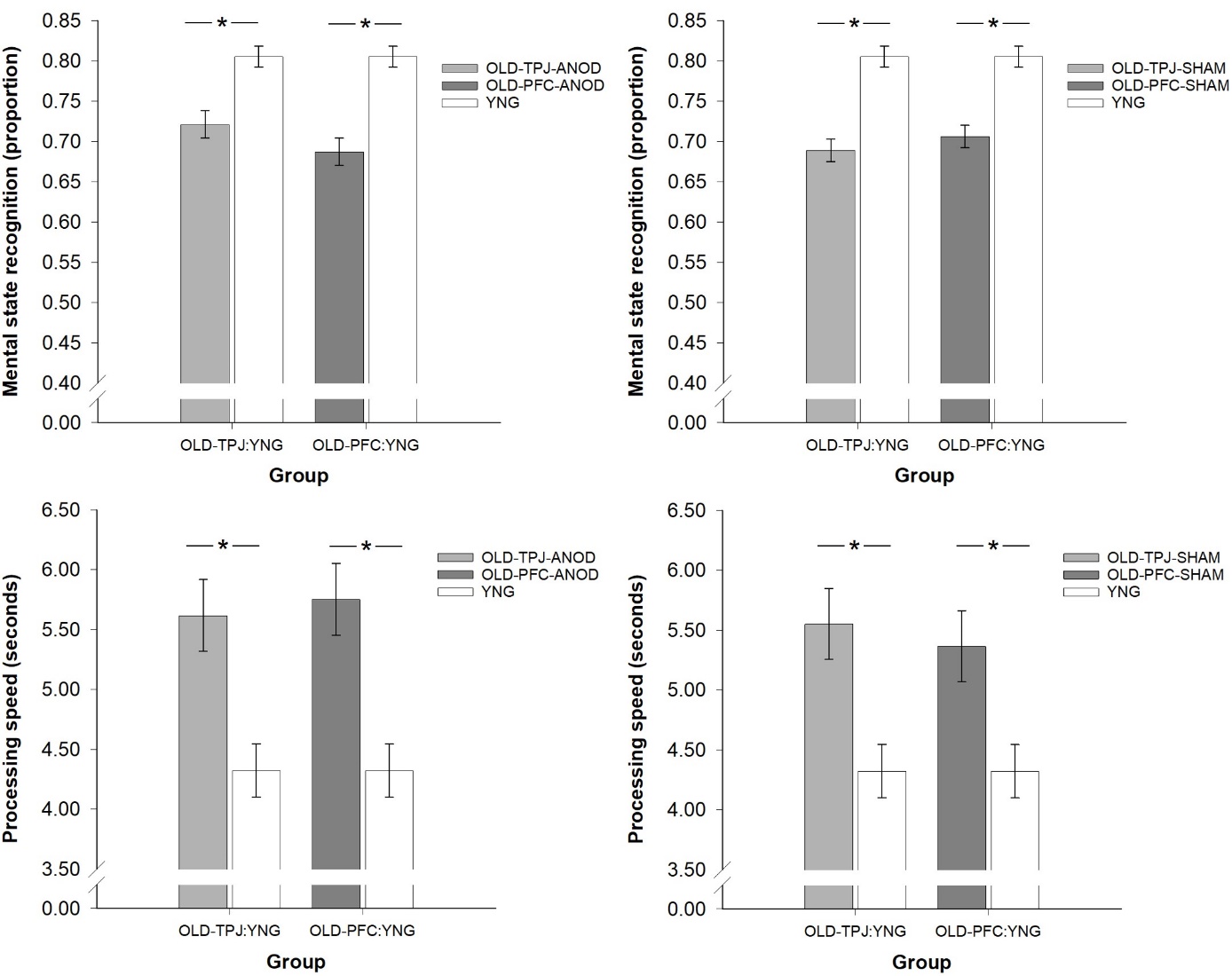

Supplement: Supplementary file 1 — Supplementary file1 (DOCX 309 KB) [file 11357_2024_1206_MOESM1_ESM.docx]
